# Applications of Advanced Nanomaterials in Sensor Devices

**DOI:** 10.3390/ma15248995

**Published:** 2022-12-16

**Authors:** Huacheng Zhang

**Affiliations:** School of Chemical Engineering and Technology, Xi’an Jiaotong University, Xi’an 710049, China; zhanghuacheng@xjtu.edu.cn

Nanomaterials can be classified into diverse categories according to their various physical and chemical properties, dimensionality, production procedures, compositions, and homogeneity. Particularly, due to the possession of nanoscale dimensions and high surface-to-volume ratio, advanced nanomaterials have the capacity to show a series of exceptional properties, such as chemical, mechanical, optical, and magnetic ones. Very recently, researchers began to pay a lot of attention to the design and synthesis of diverse nanomaterials, such as organic, inorganic, and organic–inorganic hybrid ones with controllable geometry, morphology, and topology, and aimed to explore various academic and industrial applications, such as sensing devices. For example, they have been taking advantage of the reasonable design in molecular engineering with regard to a rising star in macrocycle and supramolecular chemistry—pillar[n]arene. With its rigid cylindrical crystalline structure and nano-sized hydrophobic electron-rich cavity, as discovered by Ogoshi Tomoki in 2008, it has the capacity to mimic the chemical architecture of sounded inorganic material. This also applies to carbon nanotubes (CNTs), which are very popular for fabricating diverse marvelous sensing devices [1]. 

Thus, the design and synthesis of diverse novel organic–inorganic hybrid nanomaterials, as well as fully utilizing traditional inorganic materials, provides the opportunity to enlarge the family of sensor devices. For example, Lu and Tai et al. [2] designed and prepared the water-stable luminescent Zn^II^-metal organic framework (MOF) with the three-dimensional (3D) (2,3,8)-connected (4^3^)_2_(4^6^.6^6^.8^15^.12)(8) topological framework, as characterized by single crystal X-ray diffraction based on H_2_PBA and 1,10-phenanthroline under solvothermal conditions. Interestingly [2], the following luminescence-sensing studies indicated that such hybrid architecture could be further developed as a multifunctional material for detecting metal cations such as Fe^3+^/Cu^2+^, pollutants such as trinitrophenol, as well as drugs such as colchicine, which may pave the way towards applying MOFs to chemical sensors. 

Additionally, processing, morphological control, as well as physiochemical properties of nanomaterials have always affected each other’s performances. Thus, the academic research on morphological control over nanomaterials could efficiently promote their physiochemical properties for sensing. For example, water-soluble pillar[n]arene could efficiently promote the aqueous solubility of single-walled CNTs through sounded π−π stacking interactions for further stabilization of silver and gold nanoparticles [1]. Thus obtained organic–inorganic nanomaterials exhibited interesting applications to electrochemical sensing high toxic herbicides, as well as pollutes such as *p*-dinitrobenzene.

For future studies and developments in this flourished area of “applications of advanced nanomaterials in sensor devices”, firstly, researchers should continue to explore new advanced materials such as metal oxides and hybrid materials, while developments might pay attention to fabricating practical devices such as MEMS/NEMS and emerging semiconductors. Secondly, scientists might also focus on the mechanisms of thus-obtained sensor devices, such as “details behind the scenes”, which is a device often used in mysteries or science fiction. Theoretical investigations will guide the future direction in this area for not only enlarging the family of nanomaterials but also enhancing performances of current sensor devices.
